# Mining the red deer genome (CerEla1.0) to develop X-and Y-chromosome-linked STR markers

**DOI:** 10.1371/journal.pone.0242506

**Published:** 2020-11-23

**Authors:** Krisztián Frank, Nóra Á. Bana, Norbert Bleier, László Sugár, János Nagy, Júlia Wilhelm, Zsófia Kálmán, Endre Barta, László Orosz, Péter Horn, Viktor Stéger

**Affiliations:** 1 Agricultural Biotechnology Institute, National Agricultural Research and Innovation Center, Gödöllő, Hungary; 2 Department of Animal Breeding Technology and Management, Faculty of Agricultural and Environmental Sciences, Kaposvár University, Kaposvár, Hungary; 3 Ministry of Agriculture, Department of Wildlife Management, Budapest, Hungary; 4 Department of Wildlife Biology and Ethology, Faculty of Agricultural and Environmental Sciences, Kaposvár University, Kaposvár, Hungary; 5 Game Management Landscape Center, Faculty of Agricultural and Environmental Sciences, Kaposvár University, Bőszénfa, Hungary; 6 Department of Biochemistry and Molecular Biology, Faculty of Medicine, University of Debrecen, Debrecen, Hungary; 7 Department of Genetics, Faculty of Science, Eötvös Loránd University, Budapest, Hungary; Universite de Liege, BELGIUM

## Abstract

Microsatellites are widely applied in population and forensic genetics, wildlife studies and parentage testing in animal breeding, among others, and recently, high-throughput sequencing technologies have greatly facilitated the identification of microsatellite markers. In this study the genomic data of *Cervus elaphus* (CerEla1.0) was exploited, in order to identify microsatellite loci along the red deer genome and for designing the cognate primers. The bioinformatics pipeline identified 982,433 microsatellite motifs genome-wide, assorted along the chromosomes, from which 45,711 loci mapped to the X- and 1096 to the Y-chromosome. Primers were successfully designed for 170,873 loci, and validated with an independently developed autosomal tetranucleotide STR set. Ten X- and five Y-chromosome-linked microsatellites were selected and tested by two multiplex PCR setups on genomic DNA samples of 123 red deer stags. The average number of alleles per locus was 3.3, and the average gene diversity value of the markers was 0.270. The overall observed and expected heterozygosities were 0.755 and 0.832, respectively. Polymorphic Information Content (PIC) ranged between 0.469 and 0.909 per locus with a mean value of 0.813. Using the X- and Y-chromosome linked markers 19 different Y-chromosome and 72 X-chromosome lines were identified. Both the X- and the Y-haplotypes split to two distinct clades each. The Y-chromosome clades correlated strongly with the geographic origin of the haplotypes of the samples. Segregation and admixture of subpopulations were demonstrated by the use of the combination of nine autosomal and 16 sex chromosomal STRs concerning southwestern and northeastern Hungary. In conclusion, the approach demonstrated here is a very efficient method for developing microsatellite markers for species with available genomic sequence data, as well as for their use in individual identifications and in population genetics studies.

## Introduction

Microsatellites, also called Short Tandem Repeats (STRs) or Simple Sequence Repeats (SSRs) are DNA stretches made up of tandemly repeated short units not longer than six bases [1]. They are multi-allelic, co-dominant, and abundant with a wide genome coverage. Microsatellites have been extensively used as molecular markers for high-resolution assessment of genetic variation in many research areas, such as population biology, forensic genetics, wildlife conservation, phylogeography, genome mapping and the study of genealogy [1–3]. Advances in sequencing technology and bioinformatics continuously influence many fields of genetics and genomics, including methods to develop microsatellite markers. Traditional genomic library-based microsatellite development techniques are labor intensive, technically demanding, relatively costly and suffer from low efficiency [4, 5], especially in the case of non-model species with limited genomic information. The high throughput and low cost of next-generation sequencing (NGS) technologies enable the generation of large amounts of genomic sequences, from which putative microsatellite markers can be identified rapidly. NGS sequencing has been used for developing markers in plants [3, 6–8], fungi [3, 9], arthropods [10–12] as well as in vertebrates like fish [13], snakes [14, 15], birds [3, 14, 16] and mammals [2, 15]. Furthermore, previously assembled genomes can be exploited [9, 17, 18].

The Y-chromosomal STR variations that map to the non-recombining part of Y deserve particular attention due to their paternal inheritance. These STRs were exceptionally valuable for the reconstruction of population histories (e.g. [19]) including estimation of demographic parameters [20], for genealogical relationships and male lineage determination [21], mostly in humans [22].

The genus *Cervus* is widely distributed in the Holarctics. Its European member, the red deer (*Cervus elaphus* L. 1758) inhabits a wide range of environments [23–25]. Deer have cultural, ecological and an increasing economic importance. Being among the most important game animals for trophies, their populations have been managed, translocated and selectively hunted throughout their history and distribution area [26–30]. Recently a worldwide “deer industry” has been developed, whereby animals are farmed for venison and to some extent for antler products [31–33].

Genetic identification of red deer has become very important in forensic and population genetics as well as for wildlife conservation and parentage testing in animal breeding [25, 31, 34, 35]. Although microsatellite markers have been used in red deer, genetic studies mostly rely on adopted STR markers originally developed in other cervids [31, 35, 36]. To date no reports on the development of microsatellite markers for red deer utilizing NGS technology and exploiting the sequence of the red deer genome have been published.

Here, the recently sequenced genome of the red deer was used for STR marker development. The sequence reads were assembled, ordered along the deer chromosomes and annotated for some 21000 genes [37]. One main goal of this study was to demonstrate the usefulness of available genomic data to identify X- and Y-chromosome-specific microsatellites in the red deer. Thus, we selected X- and Y-chromosome-linked microsatellite loci from the red deer genome, and validated them by PCR amplification and genotyping. We believe that this approach would be of particular advantage for the characterization of microsatellite loci suitable for population, evolutionary and forensic genetics studies of non-model species as well as in breeding projects and game management. The discriminatory power of a combined set from autosomal and X/Y chromosomal STRs is also demonstrated here by its capability to distinguish two deer subpopulations in Hungary.

## Materials and methods

### The reference genome and microsatellite mining

The recently published genome of red deer [37] was used for microsatellite mining. The complete genome assembly CerEla1.0 (with sequences ordered in chromosomes and annotated) is available at the NCBI GenBank under the accession MKHE00000000. Assembled chromosomes of the red deer genome were used to detect microsatellites with the help of the Perl script QDD [38] with basic motifs with unit sizes of one to six bases (mono- to hexanucleotide repeats). The minimal repeat number required was 10, 7, 5, 3, 3 and 3 for the mono-, di-, tri-, tetra-, penta- and hexanucleotide repeats, respectively. Flanking sequence length at both ends was set as 200 bp. Primers for each locus with at least 80 bp flanking sequence on both sides were designed using Primer3 [39]. Primer design parameters were as follows: length from 18 bp to 23 bp with 21 bp as the optimum; annealing temperature between 55°C and 63°C with 60°C as the optimum; GC content from 40% to 60% with 50% as the optimum; and PCR product size between 100 and 500 bp.

### Sampling for population study

**Fig 1:**
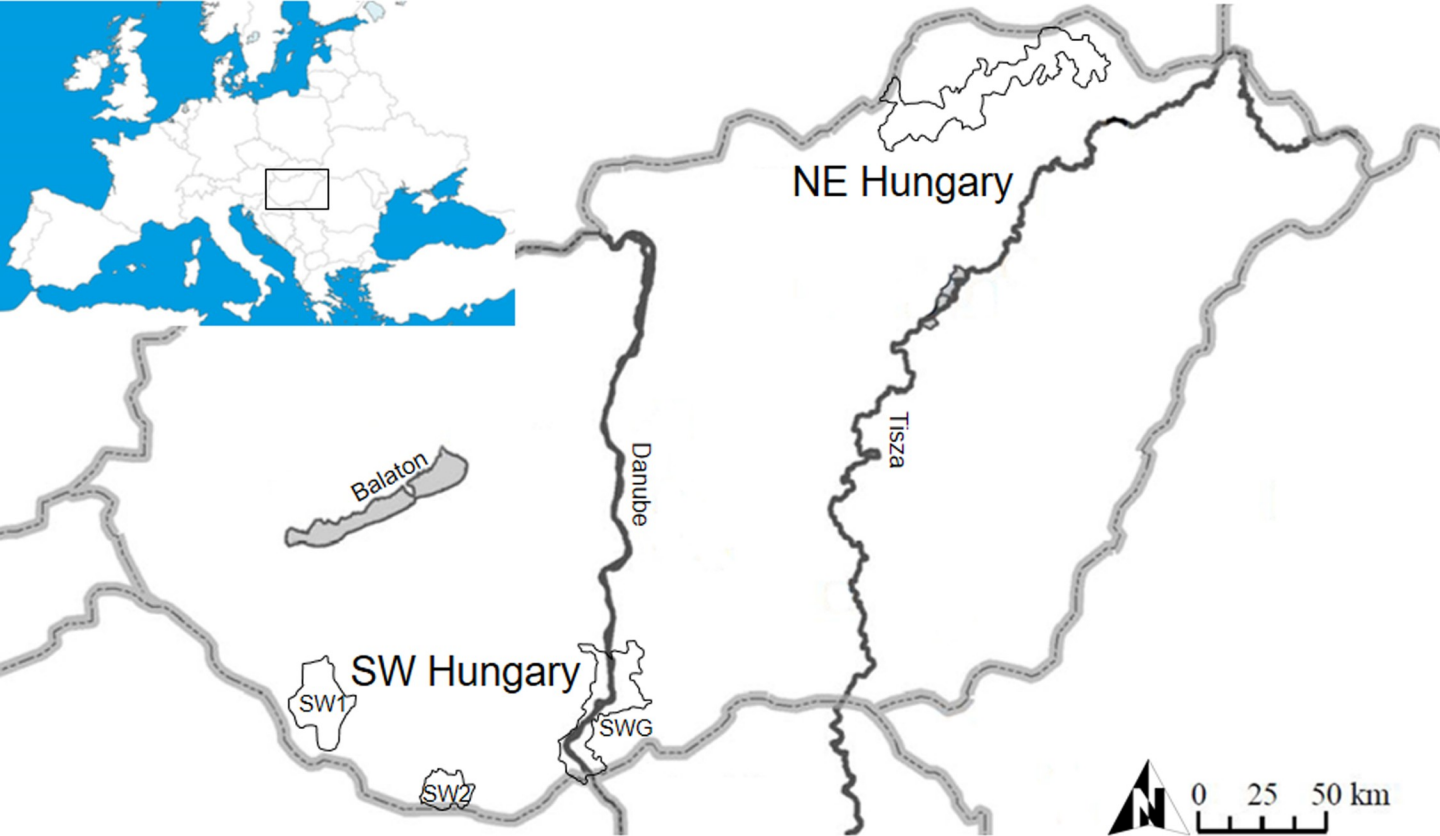
Map of the red deer sampling sites in southwestern (SW) and northeastern (NE) Hungary. Sampling sites: Lábod (SW1, n = 24), Vajszló (SW2, n = 12), Gemenc Forest (SWG, n = 51), and northeastern Hungary (NE, n = 36).

For the population analyses a total of 123 samples were collected from legally hunted red deer stags during the hunting season of 2014/2015 in Hungary; for this no specific ethical approval is needed. All applicable international, national, and/or institutional guidelines for the care and use of animals were followed. Samples were obtained from two distinct regions of Hungary, separated by historical barriers to gene flow (i.e. huge unbroken flooded lands, sandy-hill regions and steppe between and along the two big rivers, the Danube and the Tisza; later industrialization and the main rail- and highways). After the last glacial period deer migrated into the two sampling regions from different refugia [40]. Of the samples, 87 were collected in southwestern (SW) Hungary and 36 in northeastern (NE) Hungary (Fig 1). The NE sampling region comprises contiguous, unfenced hunting reserves; the SW region contains a fenced National Park (Gemenc Forest) (SWG) and hunting reserve of 180 km^2^, whereas SW1 (Lábod) and SW2 (Vajszló) are unfenced large hunting reserves. Muscle tissue samples were preserved in 96% ethanol and stored at -20°C until processing. Total genomic DNA was extracted with the Genomic DNA MiniKit Tissue (Geneaid Biotech Ltd., Taiwan) according to the manufacturer’s protocol. The quality and quantity of DNA samples were checked using a NanoDrop 1000 spectrophotometer (Thermo Fisher Scientific Inc., USA).

### X- and Y-chromosome genotyping

Eighteen sex-chromosome-linked STRs, ten for the X- and eight for the Y-chromosome were selected for amplification. The positions of these STR sites are scattered along the DNA sequence of the cognate pseudo-chromosomes of the CerEla1.0 genome assembly. Selected primers were individually tested on red deer samples for consistent amplification, and optimized for multiplex PCR. Primers were divided into two eight-plex systems, and primer concentrations were optimized for efficient amplification and fidelity. Final multiplex amplifications were performed in a total volume of 25 μl, containing 45 ng template DNA, each primer at optimum concentration (0.05–0.24 mM) and 1 × QIAGEN Multiplex PCR Master Mix (QIAGEN GmbH, Germany). A LifeECO thermal cycler (Hangzhou Bioer Technology Co. Ltd., China) was used for performing the amplifications with the following cycling conditions: initial activation at 95°C for 15 min, followed by 29 cycles of denaturation at 94°C for 30 s, annealing at 60°C for 90 s, and extension at 72°C for 60 s, with a final extension step at 60°C for 60 min.

Fluorescently labeled PCR products were separated on an ABI 3100 Genetic Analyzer (Applied Biosystems Inc., USA), using LIZ 500 (Applied Biosystems Inc., USA) as internal standard. Allele assignment was performed using Peak Scanner software (Applied Biosystems Inc., USA). For quality-control purposes, negative controls were used in each amplification, and repeated samples were used as positive controls.

### DeerPlex genotyping

Additionally, the samples were genotyped at nine autosomal and one X-chromosomal STR loci. These STRs are a component of the DeerPlex tetranucleotide STR setup (abbrev. DP), developed and published previously for forensic genetics use [35]. Initially these STRs were chosen from STR sets of wapiti (*Cervus canadensis*) and mule deer (*Odocoileus hemiomus*), then refined to the DP of red deer by the so-called zoocloning method [35, 41, 42]. The DP autosomal loci are: C01, C229, T26, T108, T123, T156, T172, T193, T501; the X-chromosomal locus is T507. Two of the autosomal STR loci (CO1 and T26) are linked, 58 Mbp apart from each other on deer chromosome 14. It is worth mentioning that all DP STRs were also found and identified in the deer genome sequence CerEla1.0. Detailed information about the STR loci and PCR composition can be found in Szabolcsi et al. [35].

PCR was compiled using the QIAGEN Multiplex PCR Kit (QIAGEN GmbH, Germany), according to the instructions of Szabolcsi et al. [35]. Amplification reactions were performed as described above for X- and Y-chromosome genotyping. Cycling conditions were as described by Szabolcsi et al. [35], except for the initial denaturation, which was adjusted to 15 minutes for the multiplex PCR kit. Separation of fluorescently labeled PCR products, allele assignments, and quality controls were as described above, in the previous section for the sex chromosomal STRs.

### Statistical analysis

Allele counts and frequencies as well as F-statistics [43] were determined by the program GenAlEx [44], which was also used for calculating expected and observed heterozygosity at each locus, Shannon’s Information Index, and gene diversity values. To interpret the effectiveness of the DeerPlex STRs, the Polymorphic Information Content (PIC) and the Probability of Identity values were calculated using the program CERVUS [45].

Due to the inheritance of the sex chromosomes, genotypes are not defined by random combination of alleles, but by the linkage of these alleles. Thus, individual genotypes of sex chromosome-linked markers were converted to haplotypes. Haplotype frequencies as well as haplotype diversity [46] values were calculated with GenAlEx [44]. Principal Coordinate Analysis (PCoA) was performed on individual multilocus genotypes by the programs GenAlEx [44] and the SYNTAX package [47], to visualize the genetic distance between the red deer stags tested. Agglomerative clustering with unweighted pair-group average (UPGMA) was applied to Euclidean distances computed between detected haplotypes with the software PAST [48].

The discriminant analysis of principal components, a multivariate method that identifies clusters of individuals without using any population genetic model [49], was used to infer the most probable number of genetic clusters. The analysis was run using the adegenet package [50] with R version 4.0.1 [51]. To determine the optimal number of genetic clusters (K) the “find.clusters” function was used with the Bayesian Information Criterion (BIC).

### Trophy evaluation

In Europe, the deer stag is one of the most important games for trophy. In trophy-centric game management the CIC formula is used internationally for evaluating the quality of trophies (CIC: Council for Game and Wildlife Conservation, www.cic-wildlife.org). The formula comprises qualitative measurements (weight in kg, lengths of main beams, brow and tray tines, circumferences at 3 points on the main beams, spread of the main beams in cm) and qualitative factors (for example, color, beading, shape of the crown); details can be seen, for example, in Bokor et al. [52]. The best trophies gain bronze (above 180 CIC points), silver (above 190 CIC points) or gold medals (“capital” antlers, above 210 CIC points). The CIC score, from a geneticist’s viewpoint, can be taken as an expression of the quantitative gross phenotype of the antlers.

For trophy scores the world ranking list [53] as well as the Hungarian Game Management Database 2015/2016 hunting years [54] was inspected. The number of “capital” trophies was counted and normalized according to hunting area and number of stags shot.

## Results

### Microsatellite mining and primer development

A total of 982,433 repeat motifs were identified at 775,019 loci in the red deer genome. We obtained 617,216 simple STR-containing sequences and 157,803 STRs in compound formation. Mononucleotide repeats (448,466) constituted the most abundant motif class (Table 1). Primer pairs were designed for 170,873 putative markers, and in this way the largest database, to date, for red deer STRs was assembled.

**Table 1:** Numbers and share of different microsatellite repeats, with the number and percentage of most abundant motif classes and maximum number of repeats in each repeat type in the red deer genome.

Repeat type	Count	% of total	Most abundant motif	Number of most abundant motif	Maximum number of repeats
Mononucleotide	448,466	45.65%	A/T	432,897 (96.53%)	96
Dinucleotide	127,243	12.95%	CA/TG	41,366 (32.51%)	83
Trinucleotide	36,804	3.75%	AGC/GCT	8426 (22.89%)	26
Tetranucleotide	256,177	26.08%	AAAT/ATTT	31,148 (12.16%)	23
Pentanucleotide	100,741	10.25%	ACTGA/TCAGT	29,175 (28.96%)	15
Hexanucleotide	13,002	1.32%	ACTTTC/GAAAGT	1435 (11.04%)	22
Total	982,433				

**Fig 2:**
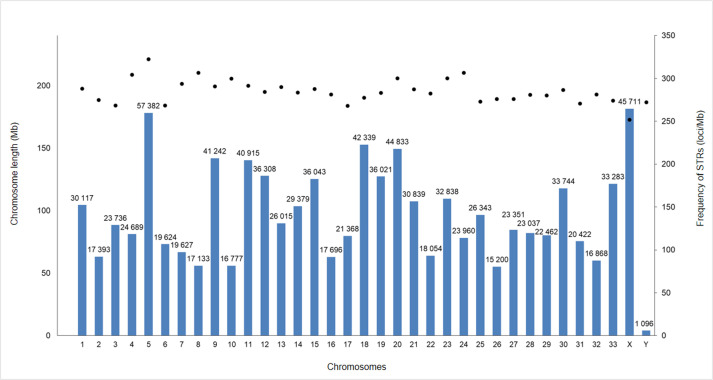
Number and frequency of microsatellite repeats in the red deer chromosomes. Left axis: Mb dimensions for chromosome lengths (bar) according to CerEla1.0 data. The number of microsatellite loci is shown at the top of each column (i.e. chromosome). Right axis: frequency of repeats as STR/Mb (dots).

The highest number of STRs were found in chromosome 5 with 57,382 loci which accounted for 5.84% of all STRs. The Y-chromosome contained the lowest number of STRs with 1096. Unlike the number of STRs, the density of STRs did not show large differences between chromosomes, it varied between 251 and 322 STR/Mb (Fig 2), whereas chromosome size was significantly positively correlated with the number of STRs (r = 0.987, P<0.0001).

### Selection and characterization of sex-chromosome-linked STRs

A total of 1779 scaffolds (i.e. sequence blocks of linked contigs, the continuous sequence runs) were mapped to the X- and 78 to the Y-chromosome. These sex-chromosome-linked scaffolds contained 45,711 STRs on the X-chromosome and 1096 STRs on the Y-chromosome. Of the X- and Y-chromosome-linked STR loci 18 were randomly selected and tested for their amplification efficiencies. Fifteen of these were successfully amplified with detectable products (Table 2), and 13 contained at least two alleles, i.e. they were polymorphic. The number of alleles per locus varied from one to six, with the average number of alleles per locus being 3.3 (Table 3). The average gene diversity value of the markers was 0.274. Gene diversity ranged from 0 to 0.670 (Table 3), with the highest value calculated for Cel_010, and zero for the two monomorphic sites Cel_002 and Cel_015. Some stags carried two separate alleles on the Cel_010 locus, and this locus amplified in hinds as well, indicating that this marker is located in the recombining region of the X-/Y-chromosomes.

**Table 2:** Characterization of 15 microsatellites/STRs located on red deer sex chromosomes.

Locus	Primer sequence (5’-3’)	Chr	Position^a^	Repeat	Plex	Conc. (mM)
Cel_001	F: GCCATCTGCCTGGTGAAG	X	175,456,980–175,457,074	GTG	1	0.07
	R: CTCATCTCTGTCCGTAAACAAGG					
Cel_002	F: TGCTTTAGGCAAATTCCTATTGT	X	179,360,960–179,361,074	TTTC	1	0.24
	R: TTTGGTCTTATCCCCACCA					
Cel_003	F: CCCTCCCTCCCTTCTTCCTT	X	13,975,991–13,976,123	TTTC	1	0.06
	R: TGTGTTCACTGAAGGATCTGTT					
Cel_004	F: TCTTCTCTCCCTCTTAGGCACA	X	116,863,960–116,864,110	TTTC	1	0.05
	R: AAGAGAGTGGAGATGTAGGTGT					
Cel_005	F: ATGCCATGCTCACGTGTCT	X	40,044,672–40,044,881	TTG	1	0.10
	R: TTGGCTAAACTCGCCTGAGC					
Cel_006	F: TTGCTGTTCTCTACCCCAGAA	X	169,092,848–169,092,949	GTAT	2	0.12
	R: AGAGATTCATTCAGTCACCAAGTA					
Cel_007	F: CCCTCTCCAGAAATAATGCTATTAACA	X	20,641,173–20,641,298	TCTA	2	0.05
	R: GCTTAGTGTAGTGCCTGGCA					
Cel_008	F: CAAGTGGTTGGTTCAGATGCT	X	175,445,165–175,445,301	CA	2	0.08
	R: TGGGAAGCCCAAATAATACCT					
Cel_009	F: TGGCTTGGCTCCATATGCAT	X	43,571,774–43,571,913	CTTT	2	0.08
	R: CCCAAAGGTGTGCTGTCTACA					
Cel_010	F: AAATCCAACAATGCTTCATCC	X/Y	160,859,594–160,859,818	CA	2	0.09
	R: CCCATGTGATCATGGTATATAATCT					
Cel_011	F: ATCTGGTTAGTCACTGTATTTCATTCC	X	170,478,980–170,479,259	AC	2	0.22
	R: AAGAACCAGCACAGCCAGATAA					
Cel_012	F: AAATAGCTGAGACATGGGAGTC	Y	2,083,464–2,083,779	AC	1	0.24
	R: CCCTGCCATACCATCAGA					
Cel_013	F: CAGGCATATTTGCATCAGAA	Y	127,045–127,440	GT	1	0.19
	R: ACCTCACCATTCTCTCACCTTC					
Cel_014	F: GAAAGCAAAATATAAATTTGAAGAACA	Y	3,202,538–3,202,955	TA	2	0.24
	R: TCCACTTCTTGGTTTTCAGAGA					
Cel_015	F: CCCCTGCAGTGGAAACAC	Y	40,309–40,768	TA	1+2	0.20
	R: CAAACCTAAACAGCACAAGCA					

^a^position of the marker in the deer genome CerEla1.0 at base pairs.

**Table 3:** Gene diversity values of the red deer sex-chromosome-linked microsatellites/STRs.

Locus	N_A_	Allele range (bp)	I	Gene diversity
Cel_001	2	95–98	0.083	0.032
Cel_002*	1	114	0.000	0.000
Cel_003	2	132–136	0.115	0.048
Cel_004	3	152–160	0.550	0.339
Cel_005	2	209–212	0.083	0.032
Cel_006	3	99–105	0.190	0.079
Cel_007	6	117–133	1.114	0.619
Cel_008	2	134–136	0.470	0.296
Cel_009	3	150–160	0.301	0.139
Cel_010	6	225–235	1.342	0.670
Cel_011	3	295–299	0.942	0.585
Cel_012	5	286–296	0.800	0.396
Cel_013	3	371–375	0.537	0.312
Cel_014	4	414–420	0.903	0.555
Cel_015*	1	457	0.000	0.000
Combined	3.267		0.495	0.274

N_A_, number of detected alleles; I, Shannon’s Information Index. Monomorphic loci are denoted with an asterisk.

**Fig 3:**
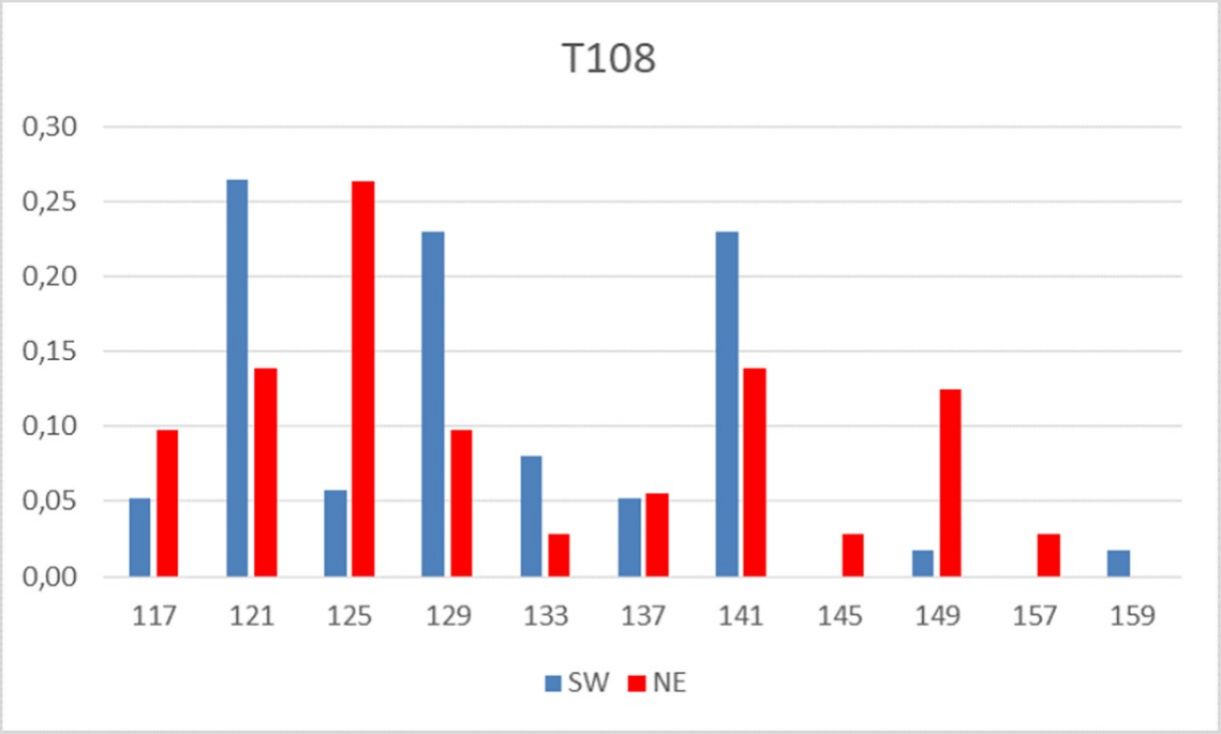
UPGMA dendrogram of the 19 Y-haplotypes found in 123 Hungarian red deer stags. The numbers of SW and NE samples belonging to the haplotypes are in brackets.

Using the Y-chromosome STRs 19 different Y-chromosome haplotypes were identified in the 123 deer stags studied (Fig 3). Six of the 19 Y-haplotypes were present in both the SW and NE regions, whereas eight haplotypes were found only in SW, and five only in NE. The most frequent Y-haplotype belonged to 50 stags (45 from SW, 5 NE); another abundant haplotype was found in 37 animals (24 from SW, 13 NE). The other haplotypes were much rarer and found only in 1–6 animals. Haplotype diversity values for SW and NE were 0.658 and 0.830, respectively. The dendrogram of Y-haplotypes showed two distinct clades. One of the branches was dominated by animals from SW: 81 of 103 (80%) samples belonged to the SW region, whereas the other, smaller branch was dominated by animals from the NE region, 14 of 20 samples (70%).

**Fig 4:**
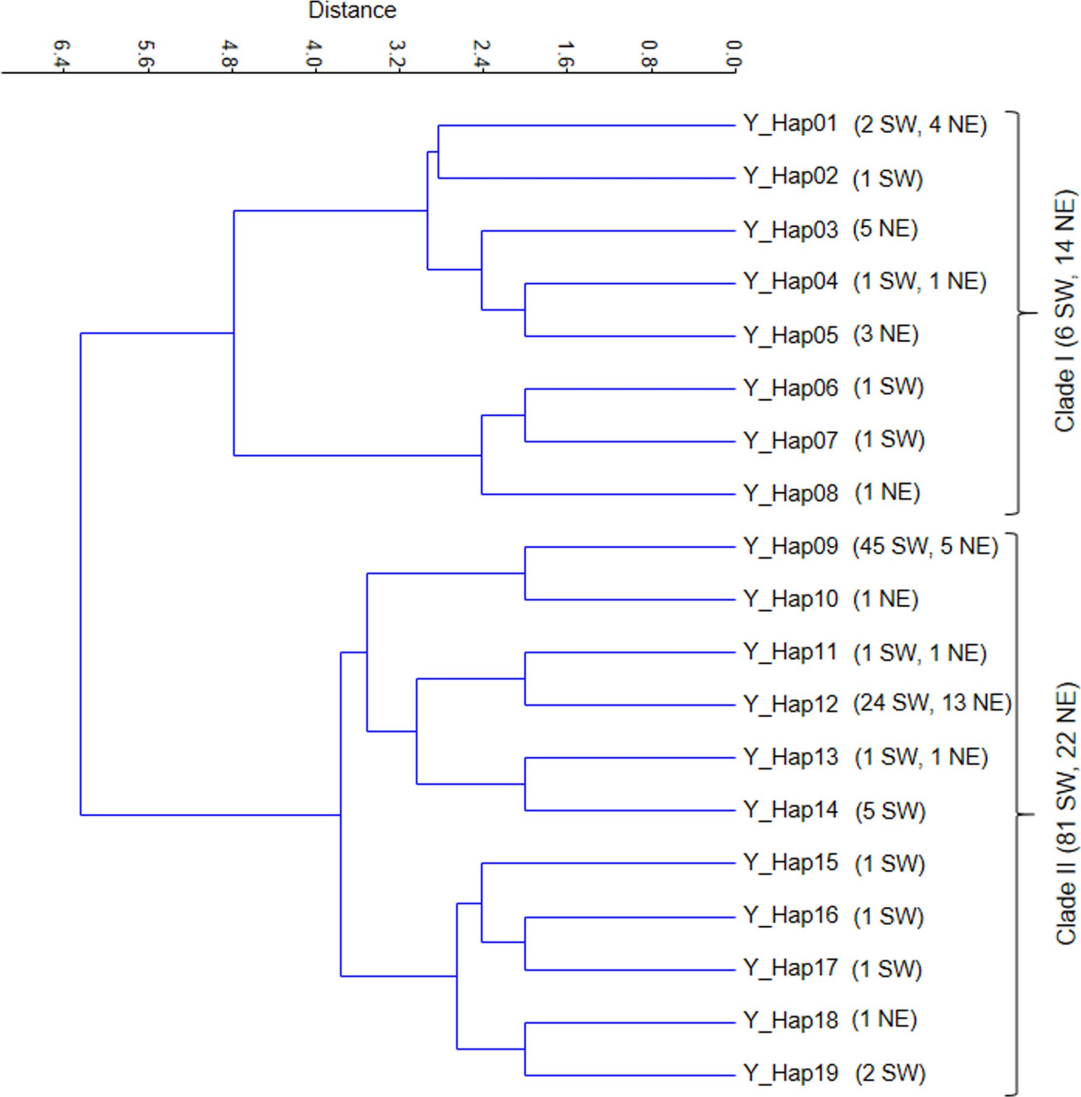
UPGMA dendrogram of the 72 X-haplotypes found in 123 red deer stags. The numbers of SW and NE samples belonging to the haplotypes are in brackets.

Using the X-chromosome STRs 72 different X haplotypes were found (Fig 4). Although the dendrogram displays two distinct X-chromosome clades, similar to the Y-chromosomes and the mtDNA inheritances, no geographical segregation is recognizable for the X-chromosome lines, and 11 of 72 X haplotypes were shared between sites.

### DeerPlex genotyping

**Fig 5:**
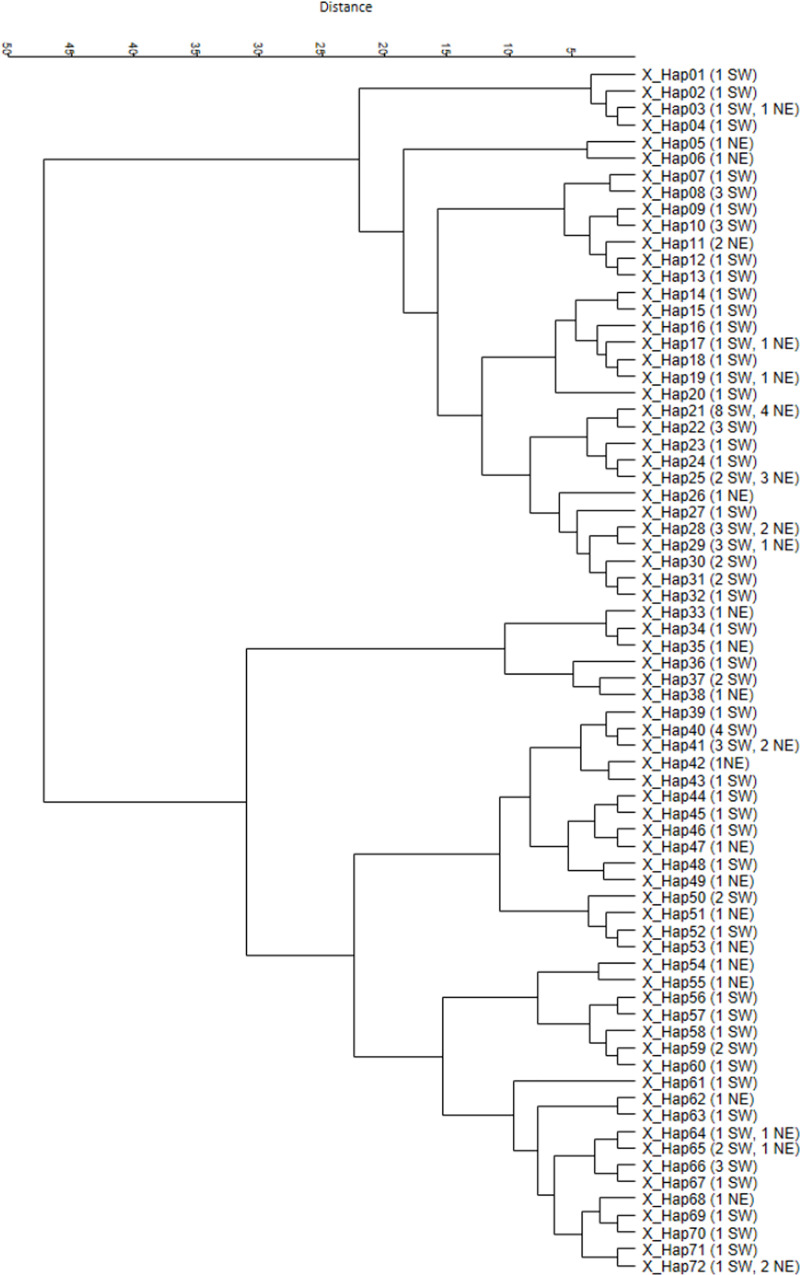
The frequencies of T108 alleles in the SW and NE populations. Numbers denote the allelic lengths in base pairs. Note the different patterns in SW vs NE.

The DeerPlex STR loci were highly polymorphic (Table 4): the number of alleles per locus ranged from six (C229) to 18 (T156), with an average of 13.7. The overall observed and expected heterozygosities were 0.755 and 0.832, respectively. Polymorphic Information Content (PIC) ranged between 0.469 (C229) and 0.905 (T193) per locus with a mean value of 0.813. Diversity values in the SW and NE populations were similarly high (Table 5). It is worth noting that the values obtained from these 123 stags are very similar to those calculated earlier from an independent sample set of 100 deer [35]. Patterns and the cognate allelic frequencies are different in SW and NE Hungary (site T108 provides a typical example, Fig 5). The F_ST_ value (i.e. the substructure parameter of the population differences) calculated is 0.030; the diversity values for the SW and NE population substructures (Table 5) and the PCoA analyses (see below) are in agreement.

**Table 4:** Genetic diversity measures for ten DeerPlex microsatellites/STRs.

Locus	N_A_	H_O_	H_E_	I	PIC	PI
C01	16	0.707	0.836	2.092	0.815	0.046
C229	6	0.415	0.500	1.031	0.469	0.281
T26	18	0.927	0.910	2.561	0.899	0.016
T108	11	0.797	0.846	2.019	0.824	0.044
T123	11	0.886	0.841	1.992	0.818	0.046
T156	18	0.724	0.880	2.357	0.865	0.027
T172	15	0.602	0.896	2.374	0.883	0.021
T193	16	0.935	0.916	2.543	0.905	0.015
T501	14	0.732	0.850	2.099	0.829	0.041
T507	12	0.829	0.842	2.104	0.823	0.041
Overall	13.7	0.755	0.832	2.117	0.813	5.88E-15

N_A_, number of detected alleles; H_O_ and H_E,_ observed and expected heterozygosity, respectively; I, Shannon’s Information Index; PIC, Polymorphic Information Content; PI, probability of identity.

**Table 5:** Genetic diversity measures averaged over ten DeerPlex STRs for the red deer subpopulations analyzed.

Region	N	N_A_	A_R_	H_O_	H_E_	I	PIC	PI
SW Hungary	87	12.9	6.44	0.755	0.815	2.024	0.792	4.743E-14
NE Hungary	36	10.6	6.90	0.756	0.832	2.031	0.805	1.316E-14

N, number of genotyped individuals; N_A_, mean number of detected alleles per locus; A_R_, allelic richness; H_O_ and H_E_, observed and expected heterozygosity, respectively; I, Shannon’s Information Index; PIC, Polymorphic Information Content; PI, probability of identity.

### Population structure

**Fig 6:**
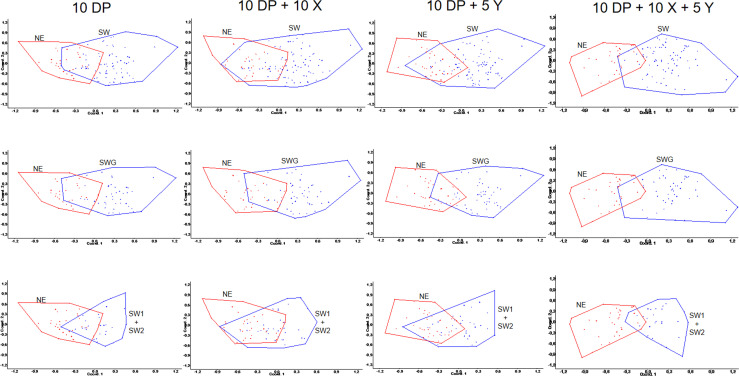
PCoAs of the 10 DeerPlex (DP), 10 DP plus 10 X-, 10 DP plus 5 Y-chromosomal and 10 DP plus 15 sex-chromosomal (i.e. combined 10 X plus 5 Y) microsatellites (STRs). Note: Combining autosomal markers with markers of the two sex chromosomes improved the discriminatory power (i.e. 1^st^ columns versus 4^th^ columns). Samples from NE Hungary are in red, SW samples in blue. SW, samples SWG, SW1, SW2 combined when compared with NE; SWG and SW1+SW2 compared with NE separately. Note the high similarity for the SW subsets (2^nd^ and 3^rd^ row).

The PCoAs shed light on the differences between the genetic structures of the NE and SW population samples, identified already at the DeerPlex STR level and further refined by the X and Y STRs (Fig 6). In concordance with the results for the autosomal, X- and Y-chromosomal haplotypes the NE and SW polygons are partially separated. This substantial but still incomplete segregation, shown by the size of the overlapping sections of the polygons, is an indication for partial admixture of the two populations.

**Fig 7:**
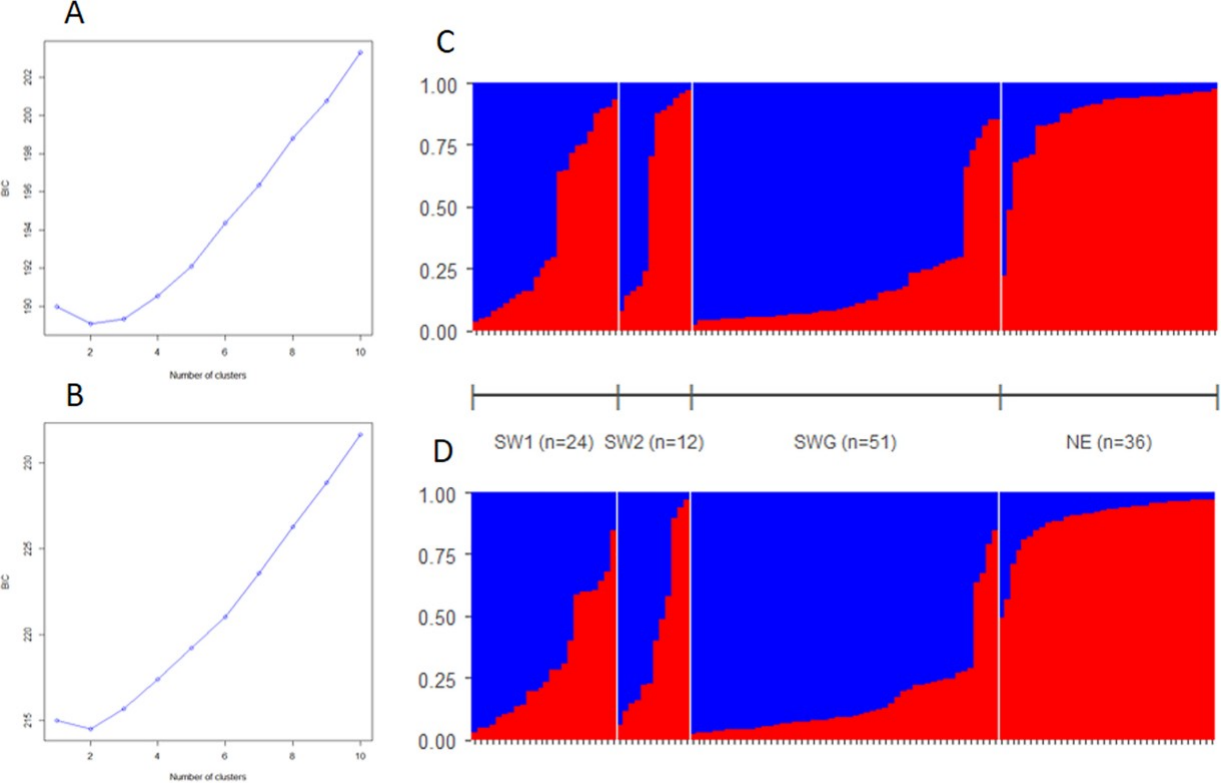
PCoAs of the 10 DeerPlex (DP), 10 DP plus 10 X-, 10 DP plus 5 Y-chromosomal and 10 DP plus 15 sex-chromosomal (i.e. combined 10 X plus 5 Y) microsatellites (STRs). (A) and (B): Bayesian information criterion (BIC) according to the number of clusters using 10 autosomal (DP) STRs and 10 autosomal (DP) plus 15 sex chromosomal STRs, respectively. Note that the most likely number of clusters is two (i.e. BIC is the lowest at K = 2). (C) and (D): Bar plot of membership probabilities at K = 2 using 10 DP STRs and 10 DP plus 15 sex chromosomal STRs, respectively. Each vertical bar represents an individual red deer with the share of probabilities assigned to the two genetic clusters (in blue and red). Samples are grouped according to sampling sites (see in brackets below the line between C and D) and aligned according membership probabilities. Membership probabilities are in blue for cluster I (“Gemenc type”), and in red for cluster II (“Zemplén type”). Note: C, average membership probabilities belonging to the cluster I in the case of 10 DP STRs are 0.585, 0.414, 0.806 and 0.135 for SW1, SW2, SWG and NE, respectively; D, in case of 10 DP plus 15 sex chromosomal STRs are 0.704, 0.646, 0.839 and 0.108 for SW1, SW2, SWG and NE, respectively.

The multivariate method of adegenet detected a genetic structure, using the ten DeerPlex STRs, as well as combining them with the 15 sex-chromosome-linked markers. The lowest BIC was obtained for a model comprising two clusters (Fig 7A and 7B). In both analyses, samples from SWG and those from NE were grouped mostly in distinct clusters with average likelihood scores of 80 and 86%, respectively. Samples from SW1 and SW2 showed some admixture (Fig 7C and 7D). In aggregate, genetic clustering indicated a separation of southwestern and northeastern populations (Figs 6 and 7). This pattern corresponds to the previous description of the presence and distribution of the European red deer mitochondrial lineages.

### Trophy quality in SW versus NE

All trophies of red deer shot legally are evaluated by the CIC formula and ranked. We inspected the world ranking list [53, 55] as well as the Hungarian Game Management Database 2015/2016 hunting years [54], and the search made it apparent that the quality of the SW trophies was much above the NE ones.

In brief: in the world ranking, among the top 300 trophies 170 were listed from Hungary, and of these 162 were from the SW region, and none from NE. The best three from NE ranked 431^st^, 548^th^ and 615^th^. In the 2015/2016 hunting years in the SW hunting resorts (17,982 km^2^) 5,377 stags were shot and their trophies evaluated. Among these, 213 were above 210 CIC points (“gold medalist”). The corresponding figures for the NE region were 10,884 km^2^, 1632 stags and only one trophy reached 210 CIC points. When these data were normalized to the resort’s area, the odds to have a “gold medalist” trophy in SW was 129-fold more in SW than in NE. Regarding the number of stags, the ratio for capital stags in the SW population was 65 times higher.

## Discussion

Next-generation sequencing technology and genome assemblies are widely used to develop STR markers for non-model species. The present study pursued two goals, namely to screen the red deer genome for microsatellites, with emphasis on sex chromosomal STRs, and to test their use in population studies, with particular interest for population structure analyses. Using genome sequence data for marker development is fast, simple, and eliminates a number of technical difficulties, compared to the traditional hybrid-capture method [1, 9, 17, 18, 56], and is now the standard approach. Here we developed a large microsatellite database for the red deer by using a genome assembly and obtained a database of putative STR markers. The previously and independently developed DeerPlex loci [35] were also searched in the red deer genome, and all loci appear in this genome-wide set. We believe that, due to the high transferability of microsatellites between related species [10, 35, 57], the newly identified markers can be used to study the origins and relationships among red deer populations, ecotypes, subspecies (i.e. wapiti, maral, sika deer) and also in other cervids like roe deer, mule deer or fallow deer. Experiences with DeerPlex are nourishing this hope, since cross reactions at nine loci (within the allelic range) were detected on fallow deer (*Dama dama*), whereas mouflon (*Ovis aries*) and cattle (*Bos taurus*) gave signals at three and one sites, respectively (however outside the allelic range), and no cross reactions were detected against roe deer (*Capreolus capreolus*), wild boar (*Sus scrofa*) and human [35].

Among the 982,433 microsatellites in the red deer genome mononucleotides were the most abundant repeat type, followed by tetranucleotides, which accounted for about 26% of microsatellites. Eukaryotic genomes are characterized by the prevalence of mononucleotide repeats over other repeat types [1, 17, 56], but our findings differed from those in previous reports, where di- and trinucleotide repeats were more frequent than tetranucleotides in vertebrates [1, 2, 17, 56, 58]. This is noteworthy because in practice tetranucleotide repeat units have proved to be more advantageous than other types of microsatellites due to technological issues like few “stutter bands”, easy discrimination of alleles, low mutation rate [59].

As expected from previous data, our results also showed that chromosome size positively correlated with the number of STRs, similarly as previously found in bovid species [56].

The majority of the newly developed sex chromosomal STR markers, 13 out of 18, showed polymorphisms, and individual genotypes were reproducible. The selected sex-chromosome-specific markers showed a relatively high gene diversity, and their frequencies of polymorphisms are comparable to those of autosomal markers [15, 16, 35]. These data reinforce previous reports that mining STRs in genome assemblies is a very efficient method of marker development [9, 17, 56].

Our previous study on the maternal (i.e. mitochondrial) lineages showed that after the last ice age red deer migrated into the Carpathian Basin from two different directions, from the West (Iberian refuge) and from the South (Balkan refuge). The corresponding maternal genetic footprints are present in today’s regional deer populations in southwestern versus northeastern Hungary [40]. Here, we tested these with nuclear microsatellites, including autosomal, X- and Y-chromosomal STRs, combined in various setups. Nine autosomal plus one X-chromosomal STR loci were taken from the DeerPlex, which had been developed previously, by the conventional method, for forensic genetics use [35]. All these STRs were also identified by the bioinformatics approach.

The potential of Y-chromosomes in population and evolutionary studies has long been recognized for providing a record of male-specific gene flow. Advances in the study of humans [19–21] and domestic animals [57, 60, 61] suggest that Y-chromosome haplotyping may develop into an important tool for studying natural populations by complementing or expanding studies using mitochondrial or nuclear markers. Our previous study on maternal (i.e. mitochondrial) lineages showed that after the last ice age, red deer migrated into the Carpathian Basin from two different directions, from an Iberian refuge and a Balkan refuge [40]. The corresponding maternal genetic footprints are present in today’s regional deer populations in southwestern versus northeastern Hungary. Results obtained in this study from Y-haplotypes agreed very well with the concept of postglacial recolonization of the Carpathian Basin, as there are two distinct Y-chromosome lineages present. This suggests that, as in the case of maternal lineages, paternal lineages also have different origins in the red deer of the region. These differences are further borne out by our previous results using autosomal STR markers; the F_ST_ values between SE and NW from the two independent STR data sets are very similar, 0.030 (this study) and 0.033 [35].

Since the stag inherits the X-chromosome from the hind, we supposed that the mtDNA lines and the X-chromosome lines show parallelisms. Although there are two X-clades, just like in the case of the mtDNA lines and the Y-chromosomes, there is no sign of the two clades themselves being concordant with the SW and NE regions. Descent lines of male X-chromosomes seem more complex and need further studies to explain, since mtDNA does not recombine, but X chromosomes do recombine in female, sex-biased dispersal, and meiotic drive might also blur genetic signatures [62].

We believe that the newly developed X- and Y-chromosome markers have the potential to provide a useful tool for recording male-specific gene flows, thus are feasible for use in population and evolutionary studies, especially when combined with mitochondrial and nuclear markers. Combinations of our newly developed markers with nuclear marker sets developed recently so far for genetic studies of red deer populations [25, 31, 35] will provide a complex toolkit for investigating their genetic structure at fine resolution. A good example, as demonstrated in our study here (Figs 6 and 7), is that with ten DeerPlex STRs supplemented with the five Y- and ten X-chromosomal STRs, the Principal Component Analyses (PCoAs) were improved, and we moved closer to our primary aim, i.e. studying correlations between geographical origins and genetic diversity of red deer in the Carpathian Basin, shown best in the case of Gemenc (SWG) vs. Zemplén (NE).

Both the SW and the NE regions provide excellent habitats for large populations of red deer, and deer do not differ in shape, size and general appearance between the two geographic regions. The only striking “phenotypic” difference, which can be extracted from the Hungarian National Committee for Evaluating Trophies Database [54] is that the SW stags develop much stronger antlers on average (i.e. heavier, thicker, with longer beams). In the top (“record”) category of trophies (i.e. “gold medalist” above 210 CIC points) the difference in frequencies is really huge, around 100-fold [53, 54]. Our findings for the SW and NE genetic profiles raise an old question to investigate: how much is the contribution of the genetic background to the phenotype and quality of the deer antler (i.e. the heritability, h^2^).

In conclusion, the approach used in this study is a powerful technique to obtain microsatellite markers from the genome of red deer, with hundreds of thousands of microsatellites identified from the assembled sequences. A large number of STR markers can be developed at the chromosome level in an efficient and effective way, as demonstrated here for the sex chromosomes. Chromosome-specific markers can then facilitate QTL (Quantitative Trait Locus) mapping, map-based gene cloning and also the integration of genetic and physical maps along with genome programs, as happened in the red deer genome program CerEla1.0. Additionally, the microsatellite database can be mined for suitable markers applicable in the study of genetic structuring of populations, as well as in game management or in forensic cases.
